# Arterial Responses in Periparturient Beef Cows Following a 9-Week Exposure to Ergot *(Claviceps purpurea)* in Feed

**DOI:** 10.3389/fvets.2019.00262

**Published:** 2019-08-08

**Authors:** Vanessa Cowan, Taylor Grusie, John McKinnon, Barry Blakley, Jaswant Singh

**Affiliations:** ^1^Veterinary Biomedical Sciences, Western College of Veterinary Medicine, University of Saskatchewan, Saskatoon, SK, Canada; ^2^Toxicology Centre, University of Saskatchewan, Saskatoon, SK, Canada; ^3^Animal and Poultry Science, College of Agriculture and Bioresources, University of Saskatchewan, Saskatoon, SK, Canada

**Keywords:** ergot alkaloid, blood flow, caudal artery, internal iliac artery, vasoconstriction, mycotoxin, hemodynamics

## Abstract

Ergot alkaloids are vasoconstrictors frequently detected in low concentrations in livestock feed. The Canadian Food Inspection Agency permits up to 3,000 μg ergot alkaloids per kg cattle feed. The objective of this study was to examine the effects of feeding low concentrations of ergot alkaloids over 9-weeks on vascular dynamics in the caudal and internal iliac arteries of beef cows. A relationship between ergot alkaloid concentration in feed and hemodynamic changes in the caudal and internal iliac arteries was hypothesized. Periparturient beef cows were randomized into four groups and group fed mixed rations containing <15 μg ergot alkaloids per kg of dry matter intake (Control, *n* = 9), 48 μg/kg (Low, *n* = 9), 201 μg/kg (Medium, *n* = 8), and 822 μg/kg (High, *n* = 6). Three experimental periods comprised the study: pre-treatment (2 weeks), treatment (9 weeks), and post-treatment (3 weeks). B-mode and Doppler ultrasonography was performed weekly to measure hemodynamic endpoints. Plasma prolactin concentrations and rectal temperatures were measured weekly. Caudal artery diameter decreased (Treatment^*^Experimental Period i.e., Tx^*^EP, *p* < 0.001) by 14% in the High group during the treatment period. Reductions (Tx^*^EP, *p* < 0.001) in caudal artery blood flow (37%, 29%) and blood volume per pulse (29%, 11%) were recorded during the treatment period in the High and Medium groups. Internal iliac artery diameter and blood flow decreased (Tx^*^EP, *p* ≤ 0.004) by 13% and 40% during the treatment period in the Medium group. Moderate reductions (Tx^*^EP, *p* ≤ 0.042; 12–25%) in the mean blood velocity during the treatment and post-treatment periods and decreases (Tx^*^EP, *p* ≤ 0.01; 12–17%) in the peak systolic velocity of both arteries during the post-treatment period were also detected. Prolactin did not change in any group during the treatment period (*p* = 0.462). Rectal temperatures were within the normal physiological range for beef cows. In conclusion, we documented moderate vasoconstriction in the caudal artery and the internal iliac artery in cows fed 201–822 μg ergot alkaloids per kg of dry matter intake for 9-week period near parturition. The pattern of alterations was similar between the caudal and internal iliac arteries. Results of this study suggest that feeding up to 822 μg/kg produce reversible pharmacological changes in beef cow vasculature and warrant reconsideration of current regulations for cattle.

## Introduction

Ergot alkaloid mycotoxins are secondary metabolites produced by the plant fungus *Claviceps purpurea* that infects cereal crops including rye, wheat, barley, triticale, and oats. Detection of ergot alkaloids in the grain and livestock feed has been on the rise in Western Canada for past 10 years (1, 2). Current Canadian Food Inspection Agency standards permit up to 3,000 μg of ergot alkaloids per kg of cattle feed (3). The ergopeptine class of ergot alkaloids are pharmacologically active compounds and are agonists of serotonergic, dopaminergic, and adrenergic receptors (4–7). Prolonged consumption of ergot alkaloids has been associated with the development of gangrenous ergotism in livestock (8–13). Gangrenous ergotism is a manifestation of ergot alkaloid toxicity marked by the progressive loss of circulation to distal limbs and tissues leading to subsequent ischemia and development of dry gangrene (8–11, 14). The principle mechanism of vasoconstriction appears to be through the agonistic activity of ergot alkaloids on smooth muscle serotonin and adrenergic receptors (6, 14–19) resulting in decreased blood flow to tissues. Despite this well-accepted pathogenesis, there is a lack of research studying the effects of subclinical low concentrations of ergot alkaloids in cattle feed within the permitted tolerance limits on the peripheral vascular system in cattle, particularly under Canadian climatic conditions.

In a previous study by our research group, beef cows were fed *C. purpurea* ergot alkaloids for 1 week and observed for signs of vasoconstriction in the caudal artery at 529 and 2,115 μg total ergot alkaloids (ergocristine, ergocornine, ergocryptine, ergosine, ergotamine, and ergometrine) per kg of dry matter (20). The caudal artery of these animals recovered to pre-treatment status once ergot was removed from the feed. It is not known if prolonged low-concentration long-term exposure to ergot alkaloids will result in cumulative and permanent alterations in vascular flow or increased tolerance at the beginning of lactation. The objective of this study was to examine the effects of feeding ergot alkaloids over a 9-week period on vascular dynamics in the caudal and internal iliac arteries of beef cows during the periparturient and early post-partum period. Based on the results of short-term study, we hypothesized a concentration-response relationship between ergot alkaloid concentration in feed and alterations in hemodynamic parameters in the caudal artery and the internal iliac artery.

## Materials and Methods

### Statement of Animal Ethics

This study was carried out in accordance with the recommendations of the University of Saskatchewan University Committee on Animal Care and Supply and Animal Research Ethics Board. The animal use protocol (#20140044) was approved by the University of Saskatchewan Animal Care Committee prior to commencing any animal work. Animals were monitored throughout the study for health and well-being using a humane intervention checkpoint. Cows were assessed for food and water intake, appearance of behavior (including signs of pain and/or distress), and vital, vasoactive, and neurological signs.

### Ration Formulation and Ergot Alkaloid Quantification in Feed

Mixed rations containing pelleted feed (barley, oat hulls, canola, and wheat screenings), barley and hay were formulated to meet the nutritional requirements of the cows using CowBytes computer program (Government of Alberta Agriculture and Rural Development, Canada). Three different concentrations of ergotized pellets were manufactured by the Canadian Feed Research Center (North Battleford, SK, Canada). Six ergot alkaloids (ergocristine, ergocornine, ergocryptine, ergosine, ergotamine, and ergometrine) were solvent extracted from feed samples and analyzed using liquid chromatography mass spectrometry as described previously (21). The lower concentration ergotized pellets (Pellet 1) contained 221 μg total ergot alkaloids per kg of pelleted feed. The intermediate pellets (Pellet 2) contained 731 μg/kg and the higher concentration pellets (Pellet 3) contained 2,981 μg/kg. The concentrations of 6 ergot alkaloids in the treatment (ergotized) pellets are given in Table 1. Control pellets contained a background of 18 μg total ergot alkaloids per kg of pelleted feed and were purchased from CO-OP® Feeds (Saskatoon, SK, Canada). Total concentrations of ergot alkaloids in feed in this study were designed to be within current Canadian guidelines for cattle, corresponding to 2,000–3,000 μg/kg total mixed ration (3). Based on the results of a previous study (20), concentrations were chosen to be conservative of development of vascular alterations as cattle were to be fed for a longer period of time than that study.

**Table 1:** Ergot alkaloid concentration (μg/kg; mean ± SEM) and percent of total (in parentheses) in the three pellet formulations.

**Ergot alkaloid**	**Ergot pellets**					
	**Pellet 1**	**(%)**	**Pellet 2**	**(%)**	**Pellet 3**	**(%)**
Ergosine	13.2 ± 0.4	(6)	46.8 ± 3.3	(6)	193 ± 6.5	(6)
Ergocornine	18.0 ± 2.5	(8)	68.1 ± 4.3	(9)	308 ± 15.2	(10)
Ergocristine	123 ± 15.8	(56)	391 ± 26.0	(53)	1,550 ± 52.3	(52)
Ergocryptine	24.3 ± 4.8	(11)	94.5 ± 5.8	(13)	397 ± 11.4	(13)
Ergotamine	41.8 ± 1.4	(19)	130 ± 5.3	(18)	529 ± 16.2	(18)
Ergometrine	0.5 ± 0.08	(<1)	1.9 ± 0.14	(<1)	7.9 ± 0.41	(<1)
Total ergot alkaloids	221 ± 45	(100)	731 ± 85	(100)	2,981 ± 171	(100)

*Ergot alkaloids were quantified in pellets using LC/MS*.

### Animal Husbandry and Experimental Design

Periparturient Hereford cows (*n* = 32) were housed in outdoor paddocks with shelter access at the University of Saskatchewan Goodale Research Farm. Calves were housed with the cows in the outdoor pens. Animals had *ad libitum* access to water, hay, and trace mineral salt blocks (CO-OP® 2:1 beef cattle range mineral block; registration no. 641098; Federated Co-operatives Limited; CO-OP® AgroCentre, Saskatoon, SK, Canada) in their pens. Cows and calves were assessed daily for general health and signs of stress. Cows were administered 3 cc of Vétoquinol Vitamin AD-500 (Vitamin A 500 000 I.U./mL, Vitamin D 75 000 I.U./mL, Vitamin E 5 I.U./mL) prior to the study. Cows were randomly assigned to one of the four ergot treatment groups: Control (*n* = 9), Low (*n* = 9), Medium (*n* = 8), or High (*n* = 6) ergot group. Sample size was different for each treatment group due to animals being excluded for various reasons, including loss of pregnancy, late pregnancy (such that it was considered an outlier), death of a cow due to uterine perforation during parturition, and death of a calf during parturition. These factors were unrelated to ergot treatment. Cows were group fed in troughs, with each group being housed in its own paddock. Group feeding was chosen due to facility constraints and practical considerations. Feed was offered daily to animals at a level of 12.7 kg dry matter per day, i.e., ~2% of body weight on a dry matter basis. The study consisted of a pre-treatment period (2 weeks, i.e., week −2 and −1), a treatment period (nine weeks, i.e, week 0 through 8, where start of ergot feeding = week 0), and a post-treatment period (three weeks, i.e., weeks 9, 10, and 11). Ergot pellets were fed from April 17 to June 19. Cows in the control, low, medium, and high ergot groups were −9 ± 6, −7 ± 5, 0 ± 5, and 3 ± 9 days *post-partum* (mean ± standard error), respectively, at the time ergot pellet feeding (where day 0 = parturition). Calving was a potential confounding factor in this experiment and was accounted for in statistical analysis (see section below). In addition, other confounding factors measured include ambient temperature inside and outside of the barn. Ambient temperatures were recorded on sample collection days with digital thermometers. The group fed cows were offered feed to consume a total daily intake of ergot alkaloids for each of the treatment groups based on the total mixed ration (TMR), 0 (control), 48 (low), 201 (medium), and 822 (high) μg/kg (based on dry matter intake). Cows were offered 3.5 kg pellets/head daily. To achieve the desired concentration of ergot alkaloids based on dry matter intake, cows in the low ergot group were offered 2.7 kg of Pellet 1 (221 μg/kg) mixed with 0.8 kg of control pellets (i.e., total of 3.5 kg pellets). The medium ergot group were offered 3.5 kg per head of Pellet 2. The high ergot group was offered 3.5 kg per head of Pellet 3. The control group was fed 3.5 kg per head of the control pellets. The pellets were spread out in concrete outdoor troughs to allow even access to all cows in the treatment group. Cows were also fed 8.5 kg dry chopped hay (grass/alfalfa mix) and 2 kg barley per head per day in their troughs in the afternoon (i.e., after pellet consumption). Mineral mix (1:1 calcium to phosphorus mineral mix, CO-OP®, Saskatoon SK; 70 g per head) was added on top of the pellets daily (at the same time as pellet consumption) in their troughs.

### Ultrasonography of the Caudal and Internal Iliac Arteries

The caudal artery and right branch of the internal iliac artery were evaluated weekly using ultrasonography as described previously (20). The caudal artery was chosen based on its previously documented sensitivity to ergot alkaloids in feed (20, 22, 23). The internal iliac artery was selected to serve as a negative control based on the results of a previous study in which no hemodynamic changes, aside from altered pulse rate, were observed in the internal iliac following 1-week exposure to ergot alkaloids in feed in beef cows (20). Briefly, the caudal artery was imaged at the fourth coccygeal vertebra, while the internal iliac artery was measured between the uterine and vaginal branches. The MyLab™Five ultrasound system (Esaote S.p.A.) with a 7.5 MHz linear-array transducer was used for transcutaneous and transrectal imaging of the caudal and internal iliac arteries, respectively. B-mode (i.e., 2D) ultrasound was used in tandem with color flow Doppler mode to capture longitudinal arterial sections for measuring arterial diameter. Video recordings (10 s) of the artery were saved for later analysis. Color flow Doppler and spectral (power) Doppler mode were used to measure spectral waveforms for each artery. A minimum of three consecutive waveforms were recorded and saved for later analysis. The ultrasonographic endpoints analyzed were arterial diameter (longitudinal section), peak systolic velocity, end diastolic velocity, mean velocity, pulsatility index, resistivity index, and pulse rate. Arterial radius, blood volume per pulse, and blood flow per minute were calculated using equations described previously (20).

### Blood Collection

Cows were restrained in a locking head gate with halter prior to blood collection. Blood was collected via jugular venipuncture into lithium-heparinized (i.e., green-gray) vacutainer tubes (BD Vacutainer, BD, Canada). The side of the neck used for blood collection alternated by week to minimize local trauma. Plasma was separated from whole blood via centrifugation (>1,500 × g) for 15 min at room temperature and stored at −20°C until further analysis.

### Enzyme-Linked Immunosorbent Assay (ELISA) for Bovine Plasma Prolactin

A commercial competitive inhibition ELISA Kit (Cloud-Clone Corp.) for prolactin (PRL) was purchased from CedarLane (CEA846BO; Burlington, ON, Canada). The assays were conducted in the Western College of Veterinary Medicine Endocrine Service Lab (Saskatoon, SK, Canada). The assay uses biotin-labeled antibody specific for bovine PRL and the company-reported detection range of the kit is 2.47–200 ng/mL (sensitivity < 0.98 ng/mL). Standards (0–200 ng/mL) were run on each plate. All samples were assayed in duplicate on the same day as per kit instructions using the provided reagents. Holstein calf serum (previously determined by the laboratory to have a high prolactin concentration) was used as reference standards (undiluted and 1:2 dilution). Inter-assay and intra-assay coefficients of variance were 26 and 11%, respectively.

### Statistical Analysis—SAS Mixed Procedure

Statistical Analysis Software (SAS) version 9.4 with Enterprise Guide 6.1 (SAS Institute, Cary NC USA) was used for all analyses. The repeated measures mixed procedure was used to test for the effect of treatment (i.e., four ergot concentrations), experimental period (i.e., pre-treatment, treatment, post-treatment), and interactions. The data were analyzed as means of each treatment group and of each experimental period. Variables analyzed included rectal temperature, prolactin concentration, and hemodynamic endpoints for the caudal and internal iliac arteries (both measured and calculated). Week of data collection was included as a repeated variable and animals were nested within treatment groups. Random factors included in the model were ambient temperature outside, ambient temperature inside the barn, pasture and month of calving. The best fit model for the data was selected based on the smallest Akaike information criteria (AICc) value from the 10 tested covariance structures (simple, compound symmetry, heterogeneous compound symmetry, Toeplitz, banded Toeplitz, Huynh-Feldt, autoregressive, heterogeneous autoregressive, ante-dependence, and unstructured). Final analysis of the data (Type 3 Test of Fixed Effects) included main effects (Tx, treatment; EP, experimental period) or interaction terms (Tx^*^EP). Statistical significance was considered to be *p* < 0.05 (α = 0.05). Multiple comparisons were conducted where applicable using the differences of least square means. The syntax used for the final analysis was as follows:

ODS graphics on;

Proc mixed covtest;

class CowID Tx Week EP CalfMonth Pasture

model Diameter = Tx|EP/DDFM = kr htype = 3;

random CalfMonth Temp_in Temp_out Pasture;

repeated Week/subject = CowID(Tx) type = ?? r rcorr;

lsmeans Tx^*^EP/pdiff = all;

run;

ODS graphics off;

quit;

^*^Note: “??” in the syntax was replaced with the name of the covariance structure for model comparisons^*^

## Results

### General

No signs of ergot toxicity (i.e., lameness, hoof swelling, hoof or tail tip necrosis, hyperthermia) were observed throughout the study. Of the 32 cows in the study, 25 calves were born in April and 7 were born in May. Unilateral lameness was observed in three cows, but each case was determined to be unrelated to ergot by the attending veterinarian and all affected cows were treated with liquamycin (LA-200, ~70 ml subcutaneous injection dependent on body weight; Zoetis Inc., Kalamazoo MI USA). Lameness resolved after treatment. Average outdoor ambient temperature throughout the treatment period was 22°C (Range: 5–29°C).

### Hemodynamic Endpoints

**Figure 1:**
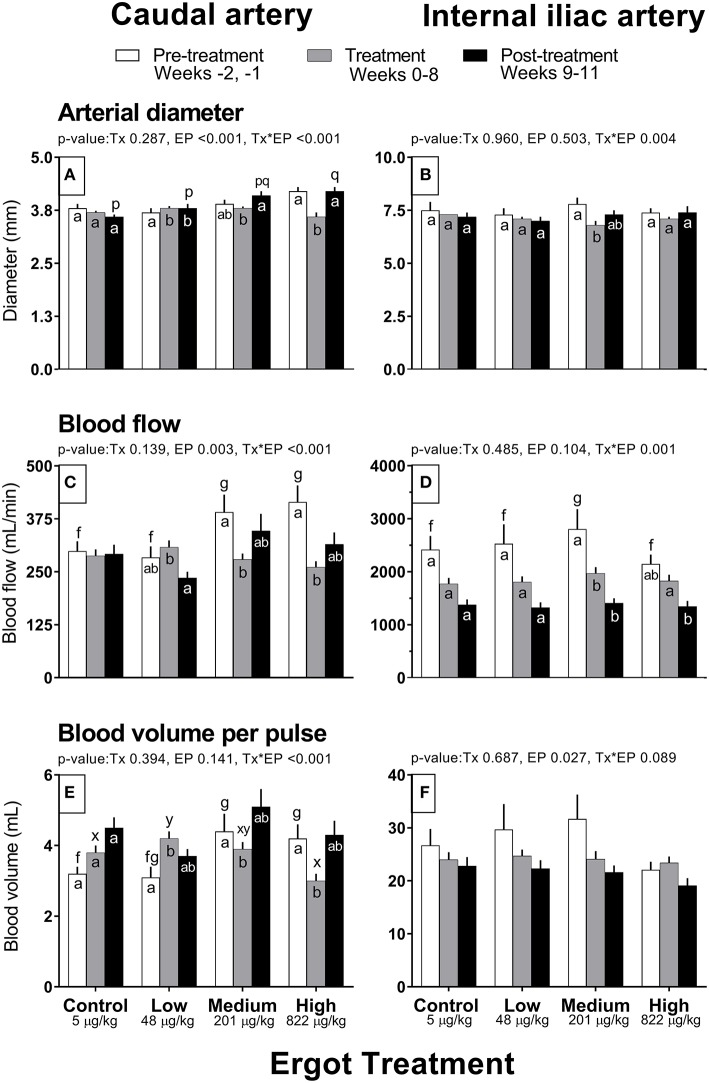
Diameter (mm), Blood flow (mL/min), and Blood volume per pulse (mL) of the caudal artery **(A,C,E)** and internal iliac artery **(B,D,F)** of periparturient Hereford cows (*n* = 32) before (2 weeks), during (8 weeks), and after (3 weeks) feeding increasing concentrations of ergot alkaloids in Control (<5 μg/kg dry matter intake), Low (48 μg/kg), Medium (201 μg/kg), and High (822 μg/kg) ergot groups. Each bar represented the mean ± SEM for each experimental period. Repeated measures analysis was used to test for changes in arterial diameter (each artery analyzed individually) for treatment (Tx), experimental period (EP) and their interaction (Tx^*^EP). Differences among experimental periods within a treatment group (connected bars) are indicated by f and g for white, x and y for gray, and p and q for black (*p* < 0.05) and differences among groups during a given treatment period (same colored bars) are indicated by a and b (*p* < 0.05).

**Figure 2:**
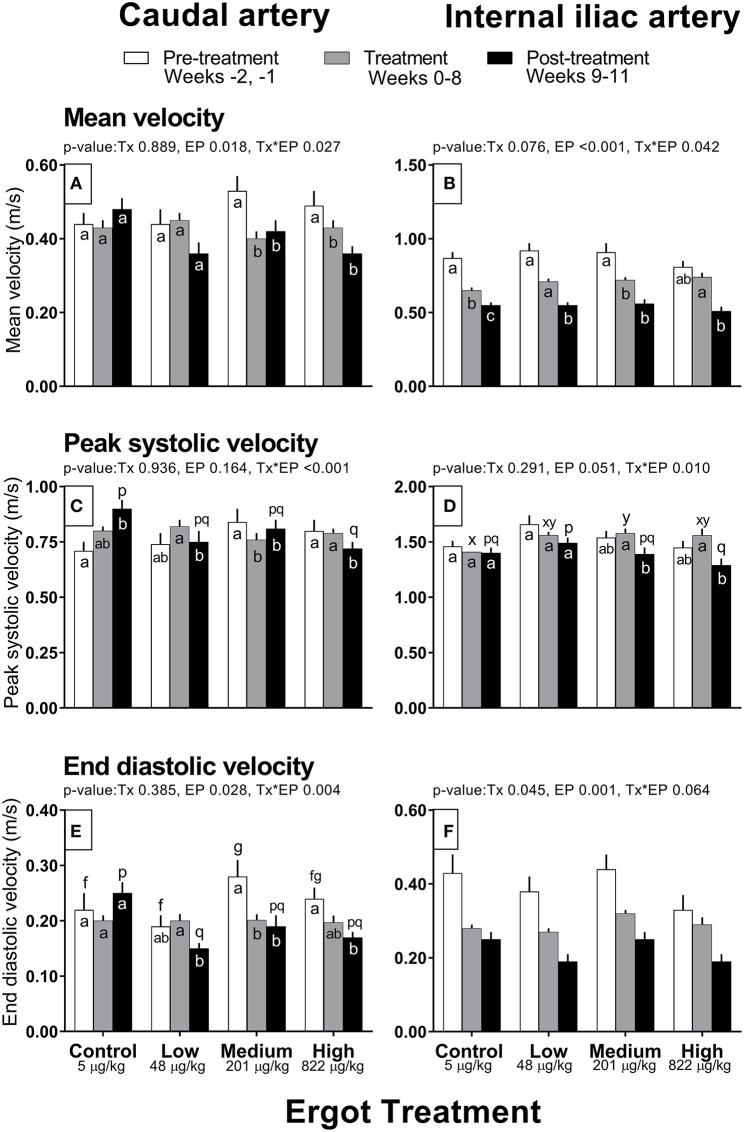
Mean velocity (m/s), peak systolic velocity (m/s), and end diastolic velocity (m/s) of the caudal artery **(A,C,E)** and internal iliac artery **(B,D,F)** of periparturient Hereford cows (*n* = 32) before (2 weeks), during (8 weeks), and after (3 weeks) feeding increasing concentrations of ergot alkaloids in Control (<5 μg/kg dry matter intake), Low (48 μg/kg), Medium (201 μg/kg), and High (822 μg/kg) ergot groups. Each bar represented the mean ± SEM for each experimental period. All velocities are in units of m/s. Repeated measures analysis was used to test for changes in arterial diameter (each artery analyzed individually) for treatment (Tx), experimental period (EP) and their interaction (Tx^*^EP). Differences among experimental periods within a treatment group (connected bars) are indicated by f and g for white, x and y for gray, and p and q for black (*p* < 0.05) and differences among groups during a given treatment period (same colored bars) are indicated by a and b (*p* < 0.05).

**Figure 3:**
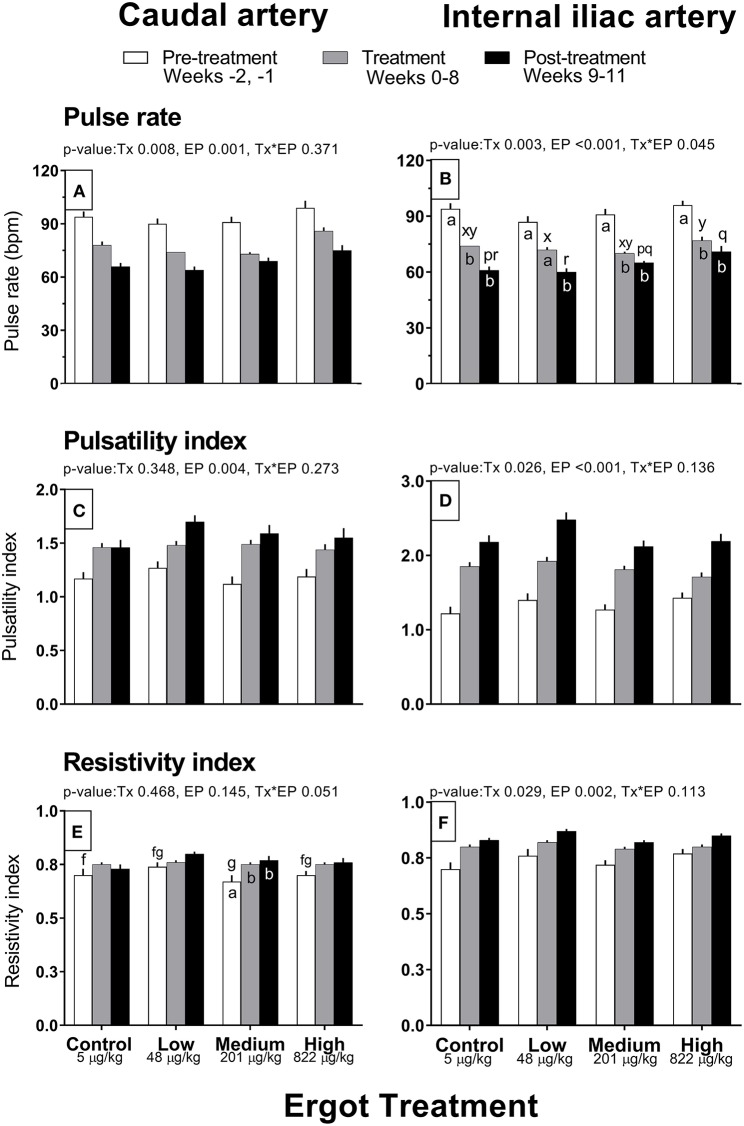
Pulse rate (bpm), pulsatility index, and resistivity index of the caudal artery **(A,C,E)** and internal iliac artery **(B,D,F)** of periparturient Hereford cows (*n* = 32) before (2 weeks), during (8 weeks), and after (3 weeks) feeding increasing concentrations of ergot alkaloids in Control (<5 μg/kg dry matter intake), Low (48 μg/kg), Medium (201 μg/kg), and High (822 μg/kg) ergot groups. Each bar represented the mean ± SEM for each experimental period. Repeated measures analysis was used to test for changes in arterial diameter (each artery analyzed individually) for treatment (Tx), experimental period (EP) and their interaction (Tx^*^EP). Differences among experimental periods within a treatment group (connected bars) are indicated by f and g for white, x and y for gray, and p and q for black (*p* < 0.05) and differences among groups during a given treatment period (same colored bars) are indicated by a and b (*p* < 0.05).

Complete data on hemodynamic parameters and variables from ultrasonographic analyses of the caudal and internal iliac arteries are presented in Supplementary Table 1. Data for arterial diameter, blood flow, and blood volume per pulse for both arteries are displayed in Figure 1. Data for blood velocities are displayed in Figure 2 and pulse rate, pulsatility index, and resistivity index are presented in Figure 3.

#### Caudal Artery Hemodynamics

##### Caudal artery diameter

In the High group, caudal artery diameter decreased on average by 0.6 mm during the treatment period (−14%) compared to the pre-treatment period (Tx^*^EP, *p* < 0.001); the diameter returned to pre-treatment value during the post-treatment period (Figure 1A). A similar pattern was observed for the Medium group where an increase in caudal artery diameter (+0.3 mm) was recorded during the post-treatment period compared to the treatment period. Caudal artery diameter increased by 0.1 mm in the Low group during the treatment and post-treatment periods compared to the pre-treatment period.

##### Caudal artery blood flow and volume

Blood flow in the caudal artery decreased (Tx^*^EP, *p* < 0.001) during the treatment period by 29% (−112 mL/min) in the Medium and by 37% (−154 mL/min) in the High groups when compared to pre-treatment period values, respectively (Figure 1C). Blood flow values during the post-treatment period were intermediate between the pre-treatment and treatment values for the Medium and High groups. Blood flow in the Low group decreased (72 mL/min; −23%) in the post-treatment period from the treatment period. Blood flow was unchanged in the Control group. Compared to the pre-treatment value, blood volume per pulse in the caudal artery also decreased (Tx^*^EP, *p* < 0.001) in the Medium (−0.5 mL; −11%) and High (−1.2 mL; −29%) groups during the treatment period and returned to pre-treatment values by the post-treatment period (Figure 1E). In the Low group, blood volume per pulse increased (1.1 mL; +26%) during the treatment period while remaining unchanged in the Control group when compared to the pre-treatment period.

##### Caudal artery blood velocities

Mean velocity was lower in the Medium and High groups during the treatment (−0.13 and −0.06 m/s, respectively) and post-treatment periods (−0.11 and −0.13 m/s) when compared to the pre-treatment values (Tx^*^EP, *p* = 0.027; Figure 2A). Likewise, peak systolic velocity and end diastolic velocities also decreased (Tx^*^EP, *p* ≤ 0.004 for both endpoints) during the treatment (−0.08 and −0.08 m/s, respectively) and post-treatment (−0.03 m/s and −0.09 m/s) periods in the Medium group but during the post-treatment period only (−0.08 and −0.07 m/s) in the High group (Figures 2C,E). Both peak systolic and end diastolic velocities were lower during the post-treatment in the Low group when compared to the treatment period (−0.07 and −0.05 m/s). In contrast, peak systolic velocity during the post-treatment period was higher (+0.19 m/s) than the pre-treatment value in the Control group.

##### Caudal artery pulse rate, pulsatility index, and resistivity index

Pulse rate of the caudal artery differed by experimental period (*p* = 0.001; 93 ± 2, 77 ± 1, and 68 ± 1 bpm in the pre-treatment, treatment, and post-treatment periods, respectively) and treatment group (*p* = 0.008; 78 ± 1, 74 ± 1, 75 ± 1, and 86 ± 2 bpm in the Control, Low, Medium, and High groups), but no interaction was detected (Figure 3A). Average pulse rate of the High group was higher than all other groups. There was decreasing trend in pulse rate for all groups throughout the experiment. Pulsatility index increased progressively over the experimental period (*p* = 0.004; 1.19 ± 0.03, 1.47 ± 0.02, and 1.58 ± 0.04 in the pre-treatment, treatment, and post-treatment periods) but did not differ among groups (Figure 3C). Resistivity index in the Medium group increased (Tx^*^EP = 0.05) by 11 and 13% during the treatment and post-treatment periods compared to the pre-treatment period (Figure 3E). Similar trends were observed in the other treatments but were not statistically different.

#### Internal Iliac Artery Hemodynamics

##### Internal iliac artery diameter

Internal iliac artery diameter decreased (Tx^*^EP, *p* = 0.004) by 13% (−1.0 mm) in the Medium group during the treatment period compared to the pre-treatment period (Figure 1B). The post-treatment value in the Medium group was intermediate between the pre-treatment and treatment values. Artery diameter remained unchanged in all other groups.

##### Internal iliac artery blood flow and volume

Blood flow decreased (Tx^*^EP, *p* = 0.001) by 40% in the Medium group from the pre-treatment to treatment period and remained reduced in the post-treatment period (Figure 1D). Blood flow decreased by 26% from the treatment to the post-treatment period in the High group. Internal iliac artery blood volume per pulse (Figure 1F) decreased over the experimental period (EP, *p* = 0.027) and was 28 ± 2, 24 ± 1, and 22 ± 1 mL per pulse during the pre-treatment, treatment, and post-treatment periods (averaged among treatment groups), respectively.

##### Internal iliac artery blood velocities

Compared to the pre-treatment period, mean velocity decreased (Tx^*^EP, *p* = 0.042) in the treatment period for the Control (−0.22 m/s) and Medium (−0.19 m/s) groups (Figure 2B). Further, the mean velocity decreased from pre-treatment to post-treatment periods in the Control (−0.32 m/s) and Low (−0.37 m/s) groups. Mean velocity decreased from treatment to post-treatment periods in the Low (−0.16 m/s) and High (−0.23 m/s) groups. Peak systolic velocity decreased (Tx^*^EP, *p* = 0.01) from the treatment to post-treatment period in the Medium (−0.19 m/s) and High (−0.27 m/s) groups (Figure 2D). End diastolic velocity varied by experimental period (*p* = 0.001; 0.40 ± 0.02, 0.29 ± 0.01, and 0.22 ± 0.01 m/s in the pre-treatment, treatment, and post-treatment periods, respectively) and treatment groups (Tx, *p* = 0.045; 0.29 ± 0.01, 0.27 ± 0.01, 0.32 ± 0.01, and 0.28 ± 0.02 m/s in the Control, Low, Medium, and High groups, respectively) (Figure 2F).

##### Internal iliac artery pulse rate, pulsatility index, and resistivity index

Pulse rate decreased (Tx^*^EP, *p* = 0.045) between the pre-treatment and treatment periods in the Control (−20 bpm), Medium (−21 bpm), and High (−19 bpm) groups (Figure 3B). A decrease in the pulse rate was also recorded during the post-treatment (vs. the pre-treatment period) for all groups. Internal iliac artery pulsatility index differed between treatment groups (*p* = 0.026; 1.83 ± 0.05, 1.97 ± 0.05, 1.80 ± 0.04, and 1.77 ± 0.05 in the Control, Low, Medium, and High groups, respectively) and increased progressively during the experimental Period (*p* < 0.001; 1.32 ± 0.04, 1.83 ± 0.03, and 2.25 ± 0.05 in the pre-treatment, treatment, and post-treatment periods, respectively) (Figure 3D). On average, the Low group pulsatility index value was greater than the Control (+7%) and Medium (+9%) groups. Internal iliac artery resistivity index differed between treatment groups (*p* = 0.029; 0.79 ± 0.01, 0.83 ± 0.01, 0.79 ± 0.01, and 0.81 ± 0.01 in the Control, Low, Medium, and High groups, respectively) and increased progressively during the experimental period (*p* = 0.002; pre-treatment, treatment, and post-treatment RI were 0.74 ± 0.01, 0.81 ± 0.01, and 0.84 ± 0.01, respectively) (Figure 3F).

### Plasma Prolactin Concentration and Rectal Temperature

Data for plasma prolactin concentration and rectal temperature are presented in Table 2. Prolactin data were previously reported (24) but was analyzed differently (i.e., by percentage decrease from baseline; weekly values). In the present study, prolactin data was analyzed using absolute values of concentrations per animal averaged over the pre-treatment, treatment period and post-treatment periods. No difference was detected in prolactin values between the treatment groups (*p* = 0.114) or during the experimental periods (*p* = 0.841). These results mirror those of the previously published results (24). Rectal temperature data were also reported previously (24) but are presented here based on the average of pre-treatment, treatment and post-treatment periods. Rectal temperature decreased by 0.6 and 0.7°C from the pre- to post-treatment period in the Control and Low groups, respectively (*p* < 0.001). Post-treatment rectal temperature was also 0.8°C lower during the treatment period for the Control group. No differences were detected in rectal temperature between experimental periods in either the Medium or High groups.

**Table 2:** Plasma prolactin concentration (ng/mL; mean ± SEM) and rectal temperature (°C; mean ± SEM) of periparturient Hereford cows (*n* = 32) during the pre-treatment (2 weeks), treatment (9 weeks), and post-treatment (3 weeks) experimental periods to increasing concentrations of total ergot alkaloids in their feed (i.e., Control, Low, Medium, and High groups).

	**Ergot treatment**			
	**Control** (5 μg/kg DM^*^)	**Low** **(48 μg/kg DM)**	**Medium** **(201 μg/kg DM)**	**High** **(822 μg/kg DM)**
***n***	**9**	**9**	**9**	**6**
Plasma prolactin (ng/mL)	*P*-values: Tx = 0.114, EP = 0.841, Tx^*^EP = 0.462			
Pre-treatment	50.5 ± 3.5	57.2 ± 6.0	65.6 ± 8.2	54.9 ± 5.3
Treatment	45.6 ± 2.4	51.2 ± 2.3	51.6 ± 2.2	42.9 ± 2.2
Post-treatment	33.6 ± 2.2	40.1 ± 3.0	44.4 ± 3.5	38.1 ± 4.7
Rectal temperature (°C)	*P*-values: Tx = 0.630, EP <0.001, Tx^*^EP <0.001			
Pre-treatment	39.0 ± 0.1^axy^	39.2 ± 0.1^ax^	38.9 ± 0.1^y^	38.9 ± 0.1^xy^
Treatment	39.2 ± 0.1^ax^	39.0 ± 0.1^ax^	38.9 ± 0.1^x^	38.7 ± 0.1^y^
Post-treatment	38.4 ± 0.1^bx^	38.5 ± 0.1^bx^	38.8 ± 0.1^y^	38.7 ± 0.1^xy^

* *DM, dry matter. Superscripts “ab” indicate differences in columns whereas “xy” indicate differences in rows. Values with uncommon alphabets are different at p ≤ 0.05. Plasma for prolactin analysis was collected weekly and analyzed by ELISA. Rectal temperature was measured weekly by handheld digital thermometer*.

## Discussion

This study evaluated the effects of prolonged low-concentration ergot alkaloid exposure on hemodynamic responses in the caudal and internal iliac arteries of beef cows around the time of parturition. The three concentrations of ergot alkaloids (48, 201, 822 μg total ergot alkaloids per kg of dry matter intake offered) were chosen to be well below the Canadian permissible levels of 2,000–3,000 μg/kg of feed (3) and lower limit was decreased compared to our previous work (20) in order to more accurately represent and examine an on-farm feeding scenario. Concentration-dependent hemodynamic changes in the caudal artery indicative of moderate vasoconstriction were observed at the medium (201 μg/kg) and high (822 μg/kg) ergot alkaloid concentrations. Recorded reductions in caudal artery diameter (14% vs. none), blood flow (37% vs. 29%) and volume per pulse (29% vs. 11%) were more pronounced in the high than the medium treatment group. In contrast, increased resistivity index was more pronounced in the medium group vs. the high group (11% vs. none). Likewise, the diameter of the internal iliac artery and blood flow decreased (10 and 40%) during the treatment period in the medium treatment. The mean blood velocity and peak systolic velocity of the caudal and internal arteries decreased between 12 and 25% during the treatment and post-treatment periods. Changes in the internal iliac artery in the medium treatment were comparable to those in the caudal artery. Most hemodynamic endpoints in both arteries recovered to pre-treatment values following removal of ergot from feed. The exceptions to this were the blood flow velocities, internal iliac artery pulse rate, and caudal artery resistivity index. Our results clearly document a relationship between the ergot alkaloid concentration in feed and alterations in hemodynamic parameters in the caudal artery. This was partially observed in the internal iliac artery. Plasma prolactin concentrations did not change, rectal temperatures changes were within normal ranges for cattle, and no clinical symptoms of ergotism were observed during the study period. These findings indicate that hemodynamics changes are sensitive bioindicators of ergot exposure and pharmacological effect.

Commonly reported hemodynamic endpoints in various arteries of livestock exposed to ergot alkaloids (originating from cereal grain ergot, tall fescue grass or perennial ryegrass) include reduced arterial diameter (20, 25), decreased arterial luminal area (22, 23, 25–30), decreased blood flow rate (20, 22, 23, 27), and decreased blood flow volume (20, 22, 23, 25). Heart rate (i.e., pulse rate) has been reported to decrease reflexively in livestock exposed to ergot alkaloids to maintain blood pressure during vasoconstriction (11, 22, 31, 32) with subsequent recovery within ~1 week of initial exposure. In the present study, internal iliac artery pulse rate decreased over the course of the experiment. Blood flow was reduced in the medium and high ergot treated groups, with decreased diameter being recorded in the medium group, suggesting that pulse rate changes may have been compensating for vasoconstriction. As expected, changes in the pulse rate were similar between the internal iliac and caudal arteries.

The caudal artery is clinically significant to ergot poisoning as tail tip sloughing is a frequently observed symptom of gangrenous ergotism in cattle (8–11, 13). Similar vasoconstrictive alterations to those reported in the present study were also observed in the caudal artery of cows following one-week exposure to 529 and 2,115 μg ergot alkaloids/kg of dry matter intake (20). The highest concentration of ergot in feed in that study was ~2.5x higher than the highest concentration in the present study, however the degree of change in hemodynamic parameters are comparable between studies. Caudal artery diameter, blood flow, and blood volume were decreased during the one-week treatment period at 2,115 μg/kg and an intermediate response was observed in the 529 μg/kg treatment. Considering together the results of the present and previous study, there is observable peripheral arterial response between 200 and 800 μg ergot alkaloids per kg of dry matter following exposure of cows to ergot for 1 to 9 weeks followed by recovery and compensation of most of the hemodynamic parameters depending on the concentration of ergot alkaloids in the feed.

In contrast to the changes in arterial diameter, blood flow, and blood volume per pulse reported herein, other studies have not found consistent effects of treatment on blood flow velocities and indices of resistance to flow (22, 23, 25). The present study observed a general decrease of caudal artery blood flow velocities in the post-treatment period, most predominantly in the animals fed 500–800 μg ergot alkaloids per kg of dry matter. Blood velocities changes were less consistent in the internal iliac artery. Decreased velocities of flow during the treatment period were not anticipated, as velocities are expected to increase in response to decreased arterial diameter and vasoconstriction (33, 34). However, decreased flow velocity following removal of ergot from the feed could indicate compensatory mechanisms to maintain blood pressure and flow. Conversely, decreased blood flow coupled with increased resistivity index [an indicator of vasoconstriction and increase vascular impedance to blood flow (35, 36)] observed during treatment and post-treatment period at 500 ergot alkaloid concentration could indicate delayed effects of ergot. It is noteworthy that there were no concentration-dependent treatment effects on pulsatility index to accompany the changes in peak systolic velocity, similar to the effects observed in cows that consumed toxic tall fescue grass containing related ergot alkaloids [reported by Aiken et al. (22)] during the treatment period. The progressive increase in pulsatility index in all groups including control animals over the course of experiment with highest values in post-treatment period was interpreted to have originated due to unrelated metabolic changes (e.g., postpartum length) or variations in environment factors.

In contrast to effects during short-term (1-week) ergot exposure in cows (20), decreased arterial diameter and blood flow in the internal iliac artery were observed around 500 μg/kg ergot alkaloid concentration in the present study. As the internal iliac artery supplies blood to the organs of the pelvis, reduced blood flow to the uterus and placenta could negatively affect ovarian function (37) and fetal development (38). A fescue ergot alkaloid study from Poole et al. (30) found decreased uterine and ovarian artery luminal areas on days 10 and 17 of the estrous cycle in treatment heifers fed 500 μg ergovaline and ergotamine per kg feed for 63 days. However, ovarian function, as indicated by antral follicle counts, corpus luteum area, and progresterone concentration were unaffected by the fescue ergot alkaloid treatment in that study. It is interesting to note that that postpartum ovarian function was also not affected by ergot treatment in our group of animals [reported earlier by Grusie et al. (24)] and cows maintained normal pregnancy (i.e., no pregnancy loss during first 2 months of gestation during observation period). Overall, it appears that caudal artery is more responsive to ergot alkaloids than the internal iliac artery, as a greater number of hemodynamic endpoints were altered in the caudal artery. We speculate that this may be related to anatomic location and histological structure of the arteries (i.e., elastic vs. smooth muscle content, internal vs. subcutaneous location) and/or the differences in abundance of serotonin and adrenergic receptors (39–42). It is unclear why an effect of ergot was present in the medium treatment vs. the high treatment group in this study. We suspect this is related to inter-animal variability within the different treatment groups. This observation needs to be confirmed.

*In vitro* studies of bovine arteries have demonstrated that arterial contractility is present despite removal of ergot alkaloids from the treatment medium (6, 43, 44). This raises concerns regarding the withdrawal times of ergot alkaloids in tissues and persistent pharmacological effects. In the present study, indicators of vasoconstriction in the caudal and internal iliac arteries of cows recovered to pre-treatment values following removal of ergot from the feed, thereby indicating no apparent persistent effect on hemodynamic parameters.

There was no effect of ergot treatment on circulating prolactin concentration in the present study. Studies of ergot alkaloid mediated fescue toxicosis in cattle commonly cite depressed prolactin concentration in the blood as a bioindicator (11, 12, 45–49), due to the dopaminergic activity of ergot alkaloids (4, 7, 50). This has been observed in other studies of fescue toxicosis, in which ergot alkaloids, principally ergovaline, are implicated in symptoms similar to *C. purpurea* ergotism. Our results, in addition to those of previous studies from our laboratory (20, 24), do not demonstrate that prolactin response is measurable when beef cattle are fed ergot alkaloids in the range 50–800 μg per kg of dry matter. Prolactin may be a more relevant and sensitive bioindicator in pigs, horses, and potentially in dairy cattle but is not a useful diagnostic indicator for subclinical ergot exposure in beef cows.

The vasoconstrictive activity of ergot alkaloids is known to increase susceptibility of livestock to heat or cold stress (11, 47). In the present study, despite arterial constriction, rectal temperatures in ergot-exposed cattle were within normal physiological ranges (51, 52). The moderate climatic conditions of this study in addition to restricting cattle to pens during treatment period (i.e., no grazing and pasture activity) made the development of hyperthermic ergotism unlikely.

In conclusion, hemodynamic alterations suggestive of vasoconstriction was demonstrated in the caudal artery and partially in the internal iliac artery of periparturient cows exposed to up to 822 μg ergot alkaloids per kg of dry matter intake for a period of 9 weeks. These included reduced diameter, blood flow in the caudal and internal iliac arteries, changes which are consistent with an altered hemodynamic state associated with ergot exposure. Prolactin concentration and rectal temperature are not useful indicators of subclinical ergot exposure in beef cows. The results of this study suggest that the current regulatory permissible levels of ergot in cattle feed may be re-examined to account for observed subclinical hemodynamic changes.

## Data Availability

The datasets generated for this study are available upon request.

## Author Contributions

VC was responsible for conducting the research, data collection and analysis, and manuscript preparation. TG was involved in animal feeding and experimental procedures. JM contributed to ration formulation, nutritional considerations, and feeding considerations. BB was involved in ergot alkaloid concentration selection, experimental design, and consultation. JS was the principle investigator of the grant and contributed toward concept development, hypotheses formulation, experimental design, oversight of the study, statistical analyses, and manuscript preparation/revisions.

### Conflict of Interest Statement

The authors declare that the research was conducted in the absence of any commercial or financial relationships that could be construed as a potential conflict of interest.
