# Supplementary material for: Arterial Responses in Periparturient Beef Cows Following a 9-Week Exposure to Ergot (Claviceps purpurea) in Feed
Source: Front Vet Sci. 2019 Aug 8;6:262. doi: 10.3389/fvets.2019.00262 (PMC6694205; doi:10.3389/fvets.2019.00262)
Supplement: Supplementary file 1 [file Data_Sheet_1.PDF]

Supplementary Material

1 Supplementary Table

SUPPLEMENTARY TABLE 1. Hemodynamic endpoints (mean ± SEM) of caudal artery and internal iliac artery measured by Doppler ultrasonography in beef cows (*n*=32) during the pre-treatment (2 weeks), treatment (9 weeks), and post-treatment (3 weeks) experimental periods to increasing concentrations of ergot alkaloids in their feed in Control, Low, Medium and High groups. Arteries were imaged weekly and data were compared by repeated measures mixed procedure.

	Ergot Treatment			
	Control (5 µg/kg DM*)	Low (48 µg/kg DM)	Medium (201 µg/kg DM)	High (822 µg/kg DM)
n	9	9	8	6
CAUDAL ARTERY				
Diameter (mm)	p-values: Tx=0.287, EP<0.001, Tx*EP<0.001			
Pre-treatment	3.8 ± 0.1[a]	3.7 ± 0.1[a]	3.9 ± 0.1[ab]	4.2 ± 0.1[a]
Treatment	3.7 ± 0.04[a]	3.8 ± 0.05[b]	3.8 ± 0.04[b]	3.6 ± 0.1[b]
Post-treatment	3.6 ± 0.05[ap]	3.8 ± 0.1[bp]	4.1 ± 0.1[apq]	4.2 ± 0.1[aq]
Blood flow (mL/min)	p-values: Tx=0.139, EP=0.003, Tx*EP<0.001			
Pre-treatment	299 ± 23[f]	284 ± 26[abf]	391 ± 41[ag]	415 ± 39[ag]
Treatment	288 ± 15	308 ± 16[b]	279 ± 14[b]	261 ± 14[b]
Post-treatment	292 ± 22	236 ± 14[a]	347 ± 40[ab]	315 ± 28[ab]
Blood volume per pulse (mL)	p-values: Tx=0.394, EP=0.141, Tx*EP<0.001			
Pre-treatment	3.2 ± 0.2[f]	3.1 ± 0.3[afg]	4.4 ± 0.5[ag]	4.2 ± 0.4[ag]
Treatment	3.8 ± 0.2[x]	4.2 ± 0.2[by]	3.9 ± 0.2[bxy]	3.0 ± 0.2[bx]
Post-treatment	4.5 ± 0.3	3.7 ± 0.2[ab]	5.1 ± 0.5[ab]	4.3 ± 0.4[ab]
Mean velocity (m/s)	p-values: Tx=0.889, EP=0.018 Tx*EP=0.027			
Pre-treatment	0.44 ± 0.03	0.44 ± 0.04	0.53 ± 0.04[a]	0.49 ± 0.04[a]
Treatment	0.43 ± 0.02	0.45 ± 0.02	0.40 ± 0.02[b]	0.43 ± 0.02[b]
Post-treatment	0.48 ± 0.03	0.36 ± 0.03	0.42 ± 0.03[b]	0.36 ± 0.02[b]

Peak systolic velocity (m/s)	p-values: Tx=0.936, EP=0.164, Tx*EP<0.001			
Pre-treatment	0.71 ± 0.04[a]	0.74 ± 0.05[ab]	0.84 ± 0.06[a]	0.80 ± 0.05[a]
Treatment	0.80 ± 0.02[ab]	0.82 ± 0.03[b]	0.76 ± 0.03[b]	0.79 ± 0.02[a]
Post-treatment	0.90 ± 0.04[bp]	0.75 ± 0.05[apq]	0.81 ± 0.04[bpq]	0.72 ± 0.03[bq]
End diastolic velocity (m/s)	p-values: Tx=0.385, EP=0.028, Tx*EP=0.004			
Pre-treatment	0.22 ± 0.03[f]	0.19 ± 0.02[abf]	0.28 ± 0.03[ag]	0.24 ± 0.02[afg]
Treatment	0.20 ± 0.01	0.20 ± 0.01[b]	0.20 ± 0.01[b]	0.20 ± 0.01[ab]
Post-treatment	0.25 ± 0.02[p]	0.15 ± 0.01[aq]	0.19 ± 0.02[bpq]	0.17 ± 0.01[bpq]
Pulse rate (bpm)	p-values: Tx=0.008, EP=0.001, Tx*EP=0.371			
Pre-treatment	94 ± 3	90 ± 3	91 ± 3	99 ± 4
Treatment	78 ± 2	74 ± 1	73 ± 1	86 ± 2
Post-treatment	66 ± 2	64 ± 2	69 ± 2	75 ± 3
Pulsatility index	p-values: Tx=0.348, EP=0.004, Tx*EP=0.273			
Pre-treatment	1.17 ± 0.06	1.27 ± 0.06	1.12 ± 0.07	1.19 ± 0.07
Treatment	1.46 ± 0.04	1.48 ± 0.04	1.49 ± 0.04	1.44 ± 0.05
Post-treatment	1.46 ± 0.07	1.70 ± 0.06	1.59 ± 0.08	1.55 ± 0.09
Resistivity index	p-values: Tx=0.468, EP=0.145, Tx*EP=0.051			
Pre-treatment	0.70 ± 0.03[fg]	0.74 ± 0.02[f]	0.67 ± 0.03[ag]	0.70 ± 0.02[fg]
Treatment	0.75 ± 0.01	0.76 ± 0.01	0.75 ± 0.01[b]	0.75 ± 0.01
Post-treatment	0.73 ± 0.02	0.80 ± 0.01	0.77 ± 0.02[b]	0.76 ± 0.02
INTERNAL ILIAC ARTERY				
Diameter (mm)	p-values: Tx=0.960, EP=0.503, Tx*EP=0.004			
Pre-treatment	7.5 ± 0.4	7.3 ± 0.3	7.8 ± 0.3[a]	7.4 ± 0.2
Treatment	7.3 ± 0.1	7.1 ± 0.1	6.8 ± 0.2[b]	7.1 ± 0.1
Post-treatment	7.2 ± 0.2	7.0 ± 0.2	7.3 ± 0.2[ab]	7.4 ± 0.3
Blood flow (mL/min)	p-values: Tx=0.485, EP=0.104, Tx*EP=0.001			
Pre-treatment	2417 ± 260[f]	2531 ± 363[f]	2805 ± 376[ag]	2148 ± 176[abf]

Treatment	1773 ± 111	1808 ± 105	1696 ± 117^{b}	1827 ± 117^{b}
Post-treatment	1380 ± 97	1325 ± 97	1409 ± 90^{b}	1345 ± 105^{a}
Blood volume per pulse (mL)	p-values: Tx=0.687, EP=0.027, Tx*EP=0.089			
Pre-treatment	26.7 ± 3.1	29.7 ± 4.8	31.7 ± 4.6	22.1 ± 1.5
Treatment	24.0 ± 1.4	24.7 ± 1.2	24.1 ± 1.5	23.4 ± 1.2
Post-treatment	22.8 ± 1.7	22.3 ± 1.6	21.6 ± 1.3	19.1 ± 1.4
Mean velocity (m/s)	p-values: Tx=0.076, EP<0.001, Tx*EP=0.042			
Pre-treatment	0.87 ± 0.04^{afg}	0.92 ± 0.05^{af}	0.91 ± 0.06^{ag}	0.81 ± 0.04^{abfg}
Treatment	0.65 ± 0.02^{b}	0.71 ± 0.02^{a}	0.72 ± 0.02^{b}	0.74 ± 0.03^{b}
Post-treatment	0.55 ± 0.02^{cpq}	0.55 ± 0.02^{bp}	0.56 ± 0.03^{bq}	0.51 ± 0.03^{apq}
Peak systolic velocity (m/s)	p-values: Tx=0.291, EP= 0.051, Tx*EP=0.010			
Pre-treatment	1.46 ± 0.05	1.66 ± 0.08	1.54 ± 0.06^{ab}	1.45 ± 0.06^{ab}
Treatment	1.41 ± 0.03^{x}	1.56 ± 0.03^{xy}	1.58 ± 0.04^{by}	1.56 ± 0.06^{bxy}
Post-treatment	1.40 ± 0.05^{pq}	1.49 ± 0.05^{p}	1.39 ± 0.06^{apq}	1.29 ± 0.06^{aq}
End diastolic velocity (m/s)	p-values: Tx=0.045, EP=0.001, Tx*EP=0.064			
Pre-treatment	0.43 ± 0.05	0.38 ± 0.04	0.44 ± 0.04	0.33 ± 0.04
Treatment	0.28 ± 0.01	0.27 ± 0.01	0.32 ± 0.01	0.29 ± 0.02
Post-treatment	0.25 ± 0.02	0.19 ± 0.02	0.25 ± 0.02	0.19 ± 0.02
Pulse rate (bpm)	p-values: Tx=0.003, EP<0.001, Tx*EP=0.045			
Pre-treatment	94 ± 3^{a}	87 ± 3^{a}	91 ± 3^{a}	96 ± 2^{a}
Treatment	74 ± 1^{bxy}	72 ± 1^{ax}	70 ± 1^{bxy}	77 ± 2^{by}
Post-treatment	61 ± 2^{bpr}	60 ± 2^{br}	65 ± 1^{bpq}	71 ± 3^{bq}
Pulsatility index	p-values: Tx=0.026, EP<0.001, Tx*EP=0.136			
Pre-treatment	1.22 ± 0.09	1.40 ± 0.09	1.27 ± 0.07	1.43 ± 0.07
Treatment	1.85 ± 0.06	1.92 ± 0.06	1.81 ± 0.05	1.71 ± 0.06
Post-treatment	2.18 ± 0.09	2.48 ± 0.10	2.12 ± 0.08	2.19 ± 0.10
Resistivity index	p-values: Tx=0.029, EP=0.002, Tx*EP=0.113			

Pre-treatment	0.70 ± 0.03	0.76 ± 0.03	0.72 ± 0.02	0.77 ± 0.02
Treatment	0.80 ± 0.01	0.82 ± 0.01	0.79 ± 0.01	0.80 ± 0.01
Post-treatment	0.83 ± 0.01	0.87 ± 0.01	0.82 ± 0.01	0.85 ± 0.01

* DM = dry matter

Pair-wise comparisons were performed if the ANOVA p-value was ≤0.05 for the treatment (i.e., Tx; control, low, medium and high), experimental period (i.e., EP; pre-treatment, treatment, post-treatment) or treatment*experimental period interaction term (i.e., Tx*EP). Superscripts abc indicate differences in columns (among periods within a treatment). Superscripts fgh indicate differences in pre-treatment period rows; xyz indicate differences in treatment period rows; pqr indicate differences in post-treatment period rows (among treatments for a given period). Values with uncommon alphabets are different at p≤0.05
